# Reduction of K^+^ or Li^+^ in the Heterobimetallic Electride K^+^[LiN(SiMe_3_)_2_]e^–^

**DOI:** 10.1021/jacs.3c06066

**Published:** 2023-07-21

**Authors:** Nathan Davison, Paul G. Waddell, Erli Lu

**Affiliations:** Chemistry−School of Natural and Environmental Sciences, Newcastle University, Newcastle upon Tyne NE1 7RU, U.K.

## Abstract

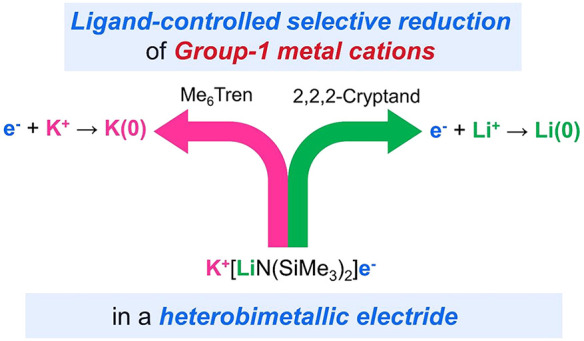

Given their very negative redox potential (e.g., Li^+^ → Li(0), −3.04 V; K^+^ → K(0), −2.93 V), chemical reduction of Group-1 metal cations is one of the biggest challenges in inorganic chemistry: they are widely accepted as irreducible in the synthetic chemistry regime. Their reduction usually requires harsh electrochemical conditions. Herein we suggest a new strategy: via a heterobimetallic electride intermediate and using the nonbinding “free” electron as reductant. Based on our previously reported K^+^[LiN(SiMe_3_)_2_]e^–^ heterobimetallic electride, we demonstrate the reducibility of both K^+^ and Li^+^ cations. Moreover, we find that external Lewis base ligands, namely tris[2-(dimethylamino)ethyl]amine (Me_6_Tren) or 2,2,2-cryptand, can exert a level of reducing selectivity by preferably binding to Li^+^ (Me_6_Tren) or K^+^ (2,2,2-cryptand), hence pushing the electron to the other cation.

Group-1 metals are the least electronegative elements in the Periodic Table, with their Pauling scale electronegativity ranging from 0.98 (Li) to 0.79 (Cs) [in comparison with the values of calcium (1.00), hydrogen (2.20), and carbon (2.55)]. As a result, zero-valent Group-1 metals intend to donate their valence shell electron and to form +1 oxidation state cations M^+^. Therefore, Group-1 metals are well-known as powerful reductants. Their coordination chemistry is dominated by the +1 oxidation state. Reducing Group-1 metal cations to zero-valent species is rather challenging, usually requiring high external electrochemical overpotential. Electrochemical reduction plays essential industrial roles in producing Group-1 metals [e.g., Li^1^ and Na (Caster process^2^)] and in Li-ion^3^/Li-metal^4^ batteries. However, the electrochemical reduction is of little use in the synthetic chemistry context: the very negative redox potentials (e.g., Li^+^ → Li(0), −3.04 V; K^+^ → K(0), −2.93 V^5^) are out of the stability window of most organic solvents; i.e., such harsh potential will unavoidably lead to severe unwanted side reactions, e.g., solvent/ligand decompositions. Beyond the electrochemical reduction, there were discrete reports of photochemical reductive coupling of organolithiums, where Li metal was postulated as byproduct but never persuasively observed.^6−11^ The photochemical mechanism is unclear, but potentially, the carbanion R^–^ donates its electron density to Li^+^ to form R· radical and fleeting Li(0) species.^12,13^

Out of the electro-/photochemical regimes, *chemical* reduction of Group-1 metal cations M^+^ to form corresponding zero-valent species M(0) is extremely scarce and is one of the toughest challenges in synthetic inorganic chemistry. A primary obstacle is the lack of an appropriate reductant to overcome the formidably negative redox potential. Very recently, the Harder group (in 2021^14^) and the Hill/McMullin groups (in 2023^15^) reported reducing Na^+^ to zero-valent Na metal, facilitated by favorable inner-sphere electron transfer from electron-rich magnesium(0)^14^ or magnesium(I)^15^ species, respectively. These two remarkable breakthroughs still have two limits to overcome: (1) The systems require specially designed ligands to bring together the Na^+^ and Mg(0)/Mg(I) fragments to facilitate the electron transfer. (2) The reductants, i.e., the Mg(0)/Mg(I) fragments, are still specialized, in spite of the expanding library and applications of Mg(I) coordination complexes.^16^

Beyond reducing a Group-1 metal cation, an even more formidable but less mentioned challenge is: Would it be possible to *selectively* reduce Group-1 metal cations (e.g., Li^+^ vs K^+^) within a heterobimetallic system? The target would be exceedingly difficult, if not impossible, for traditional routes such as tuning of the reductants, given that their redox potentials only differ by 0.1–0.3 V from Li^+^ to Cs^+^. A brand-new strategy must be developed to achieve this target.

Herein, we report the proof-of-concept of an unexplored approach to demonstrate the selective reduction of K^+^ or Li^+^, employing the nonbinding “free” electrons in the Li-K heterobimetallic electride K^+^[LiN(SiMe_3_)_2_]e^–^ (**1**)^17^ as the reductant, and different cation-binding preferences of multidentate ligands (2,2,2-cryptand and Me_6_Tren) as the tuning handle.

Before moving onto our findings, we would like to introduce a class of highly unusual negative oxidation state Group-1 metal complexes, namely, alkalide, which feature M^–^ centers (M: Na, K, Rb, Cs).^18−30^ These species are prepared by treating zero-valent Group-1 metals with chelating reagents/solvents (L), following a disproportionation route, i.e., 2M(0) + *n*L → [M^+^(L)_*n*_][M^–^]. The formation of heterobimetallic alkalides,^23,27−29^ i.e., M(0) + M′(0) + *n*L → [M^+^(L)_n_][M′^–^] (M, M’: two different Group-1 metals), may be considered, with a stretch, as a formal reduction of M′(0) by M(0). But these reactions are well-accepted as disproportionation instead of reduction.

Electride is a unique class of materials featuring nonbinding “free” electrons, which do not bind to any nucleus but are confined in space topology structures (e.g., lattice, solvent cage).^31−34^ The very low electronegativity of zero-valent Group-1 metals enables facile formation of electride phases such as Cs^+^(18-crown-6)_2_e^–^^35^ and sodium-liquid ammonia Na^+^(NH_3_)_*n*_e^–^.^36,37^ Despite enormous research interest from the physics/physical chemistry/material sciences communities for their physicochemical properties derived from the nonbinding electrons and their topologies,^31−34^ electron recombination in electrides, i.e., formal reduction of the Group-1 metal cations, still remains largely unexplored. In 2022, the Thomas group reported preparing Li metal dendrites by evaporating liquid ammonia from Li^+^(NH_3_)_*n*_e^–^.^38^ The resultant Li metal dendrites were reported to feature large surface areas and function as a reductant in chemical synthesis.

Recently, we reported a facile and scalable synthesis of a new class of room-temperature stable heterobimetallic electride, K^+^[LiN(SiMe_3_)_2_]e^–^ (**1**).^17^ The accessibility of **1** allows for a comprehensive exploration of its reactivity. From the ligand perspective, our group has applied a number of multidentate neutral ligands in Group-1 metal chemistry^39−44^ and has observed their cation-binding selectivity.^45^ Since **1** features a nonbinding electron and two Group-1 cations (Li^+^ and K^+^), it offers an unprecedented opportunity to probe selective reduction of Li^+^ or K^+^. We are intrigued by a simple hypothesis: Would it be possible to use external ligands to tune the electron’s direction (Scheme 1)?

**Scheme 1:**
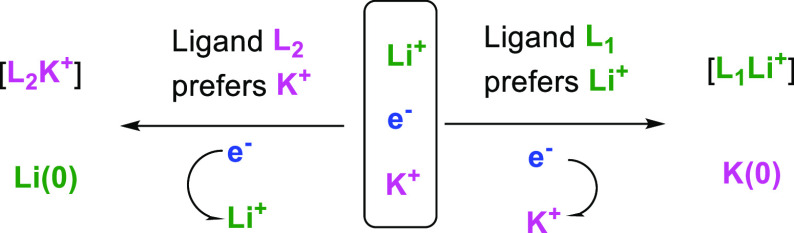
Hypothesis of Ligand-Controlled Selective Reduction of Li^+^ or K^+^ in a Li^+^-K^+^ Heterobimetallic Electride

Following the hypothesis, 2,2,2-cryptand and Me_6_Tren are the ligands of our choice. 2,2,2-Cryptand is well-known for its matched cavity size to sequester K^+^,^46,47^ although coordination with Li^+^ is also known.^48^ In comparison, Me_6_Tren prefers coordinating Li^+^;^45^ albeit, K^+^-Me_6_Tren complexes are know in the literature.^49^

Electride **1** reacts with or is insoluble in most organic solvents.^17^ Hence, the reactions between **1** and 2,2,2-cryptand/Me_6_Tren were heterogeneous and resulted in silvery metal pieces (Figure 1; see Supporting Information for details). Other than the metal pieces, after workup, coordination complexes, [Li{N(SiMe_3_)_2_}(Me_6_Tren)] (**2**) or [K(2,2,2-cryptand)][N(SiMe_3_)_2_] (**3**), were isolated in 78% and 16% crystalline yield, from the Me_6_Tren or 2,2,2-cryptand reaction, respectively (Figure 1). It should be noted that **3** is the only isolable coordination complex from the 2,2,2-cryptand reaction. We attribute **3**’s low (but reproducible) yield to two major reasons: (1) 2,2,2-Cryptand is known to be unstable toward electrides via reductive C–O/C–H bond cleavages, which promoted the Dye group to develop its specialized per-aza cryptand-analogue TriPip222.^50^ (2) **3** is not stable by itself.^51^ Removing volatiles from the crystallization mother liquor of **3** produced an intractable oily mixture, which likely resulted from the above-mentioned decompositions. Single crystals of **2** and **3** were grown from an *n*-hexane (for **2**) or Et_2_O (for **3**) solution, respectively. Both **2** and **3** are known complexes, and their crystallographic cell parameters and bond lengths/angles match with the previous reports (**2**,^52^**3**^51^).

**Figure 1:**
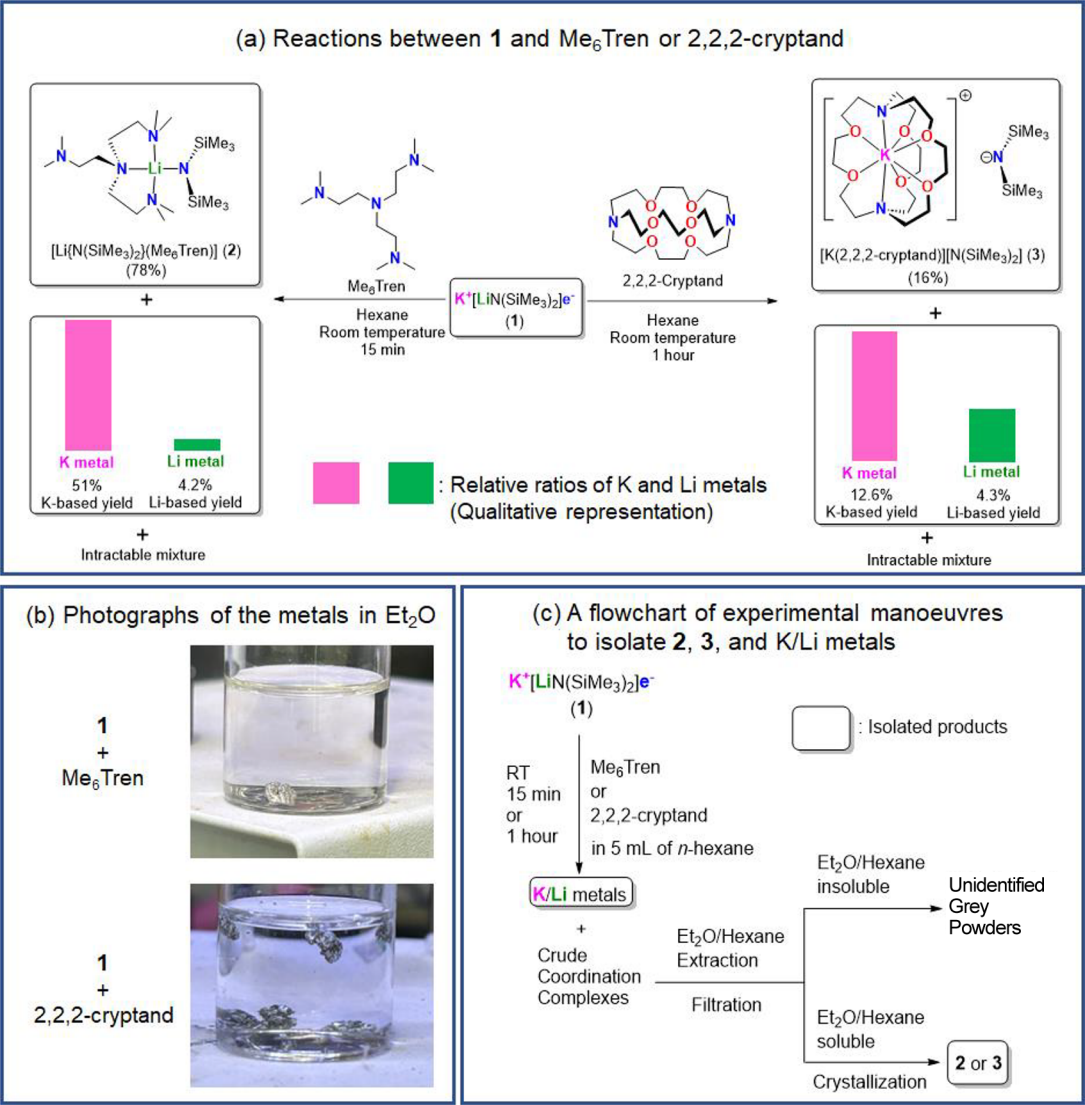
(a) Schematic presentation of reactions between electride **1** and Me_6_Tren or 2,2,2-cryptand. (b) Photographs of their metallic phases in Et_2_O. (c) Schematic flowchart for the isolation of coordination complexes **2**/**3** and the K/Li metals.

The metal pieces are the focus of this study. We noticed that all metal pieces isolated from the Me_6_Tren reaction are denser than Et_2_O (Figure 1b top), while there are floating pieces from the 2,2,2-cryptand reaction (Figure 1b bottom). Considering the densities of K metal (0.862 g/cm^3^), Et_2_O (0.713 g/cm^3^), and Li metal (0.534 g/cm^3^), it is obvious that while the Me_6_Tren reaction produces mostly K metal, the 2,2,2-cryptand reaction produces a mixture of K and Li metals.

It is essential to understand the metal contents. The metal pieces were quenched using deionized water (see Supporting Information for health and safety information), and the resultant aqueous solutions were subjected to inductively coupled plasma (ICP) analysis to reveal their K or Li contents (see Supporting Information sections 1.3.1 (page S5) and 1.4.1 (page S7) for details). ICP spectroscopy is well-known for its accuracy and reliability to analyze the metal ion identity and content.^53^ Key ICP results are listed in Table 1. It should be noted that the ICP results are irrelevant to metals’ oxidation states prior to quenching; i.e., they could be contaminated by K^+^/Li^+^ impurity residue on the surfaces or wrapped in the metal pieces. To minimize the potential contamination, all of the metal pieces were thoroughly washed with Et_2_O and *n*-hexane prior to quenching.

**Table 1:** Metal Masses, the ICP Results and the Translated Metal Yields and Li Metal Molar Ratios (*m*_Li_)

	1 + Me_6_Tren (0.5 mmol)	1 + 2,2,2-cryptand (2 mmol)
Overall metal mass (mg)	10.1	10.5
	ICP Results (ppm)	
Li (*n*_Li_)	62.93	128.12
K (*n*_K_)	766.30	383.06
	The translated K and Li metal yields	
K metal yield (K-based)	51.0%	12.6%
Li metal yield (Li-based)	4.2%	4.3%
Li metal molar ratio in the metallic phase *m*_Li_ = *n*_Li_/(*n*_Li_ + *n*_K_)	0.076	0.251

For both reactions, the major component in the metallic phase is potassium, but the lithium content ratio is much higher in the 2,2,2-cryptand reaction (*m*_Li_ = 0.251) cf. the Me_6_Tren reaction (*m*_Li_ = 0.076). The relative K and Li metal contents are qualitatively visualized in Figure 1a by the heights of the magenta (K metal) and green (Li metal) bars. It is evident that Me_6_Tren selectively binds to Li^+^ and forms **2** in high yield (78%), forcing the nonbinding “free” electron to reduce K^+^ and form K metal in 51% yield with a low Li metal content (*m*_Li_ = 0.076) (Table 1). On the other hand, 2,2,2-cryptand binds to K^+^, forming the SIP complex **3** as the only isolable coordination complex in a low but reproducible yield (16%). The overall yield of the metallic phase is lower in the 2,2,2-cryptand reaction cf. the Me_6_Tren reaction: we postulate this again as a result of the 2,2,2-cryptand’s decomposition upon treatment with an electride.^31,50^ Nevertheless, from the visual evidence (Figure 1b) and the ICP results (Table 1), it is obvious that the Li metal content (*m*_Li_) is higher than that of the Me_6_Tren reaction. Hence, despite their low yields, we are confident to conclude that the formations of **3** and Li metal are interconnected: 2,2,2-cryptand binds to K^+^ and pushes the electron to reduce Li^+^. Overall, our observations can be concluded as a self-consistent scenario in Figure 2.

**Figure 2:**
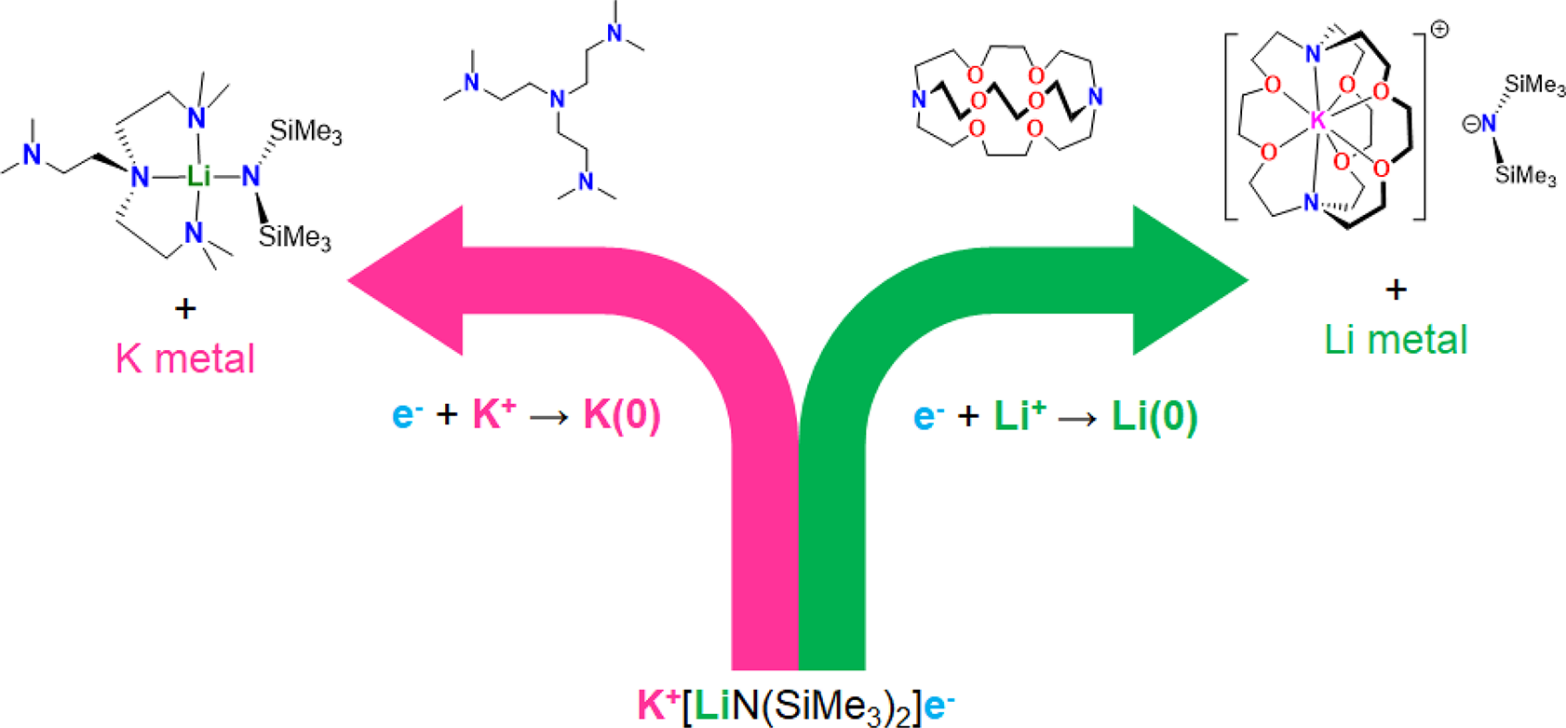
Conceptual presentation of the ligand-tuned selective reduction of K^+^ or Li^+^.

Other than Me_6_Tren and 2,2,2-cryptand, 18-crown-6 (18-c-6) was also tested as a well-known ligand to bind K^+^. However, the reaction between 18-crown-6 and one equivalent of **1** produced no observable metals. The only isolable products are [(18-c-6){KN(SiMe_3_)_2_}] (**4**) and [(18-c-6)(K)][Li{N(SiMe_3_)_2_}_2_] (**5**) (see section 1.5 in Supporting Information). Due to their similar solubilities and crystallizability, **4** and **5** co-crystallize: their structures are identified by SCXRD, but meaningful yields are not available. It is sensible to assume that **4** and **5** are not the major products of the reaction: their combined crystalline mass is around 20 mg from a 0.5 mmol reaction (132 mg of 18-c-6, 103 mg of **1**). We presume that the low yield and less-controlled reaction are again due to the instability of 18-crown-6 with electride (similar to 2,2,2-crypyand).^31,50^

To summarize, in this work, we proved the concept of ligand-tuning selective chemical reduction of K^+^ or Li^+^ (Figure 2). Despite its low yield, the Li^+^ reduction is particularly interesting: Since the electride **1** was synthesized from K metal and [LiN(SiMe_3_)_2_],^17^ the 2,2,2-cryptand reaction herein can be considered as a formal stepwise Li^+^ reduction by K metal (Scheme 2a). From this perspective, the electride **1** can be considered as a reduction intermediate (Scheme 2b), where the nonbinding electron is a ready-for-action highly active reductant.

**Scheme 2:**
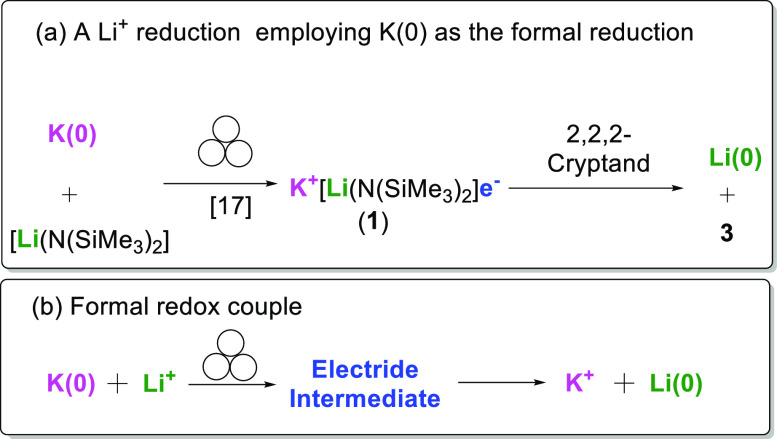
(a) Reducing Li^+^ using zero valent K metal; (b) The formal redox couple, *via* the electride intermediate

Though K or Li *metals* were isolated in this work, in principle, it is possible to trap discrete Li(0)/K(0) *molecular coordination complexes* with the appropriate ligand(s). Further work is underway in our group toward this target, utilizing the heterobimetallic electride **1** and its congeners as the gateway, to unlock zero-valent Group-1 metal chemistry. Finally, together with recent breakthroughs from other groups,^54,55^ this work highlights the unique value of mechanochemistry in synthesizing otherwise impossible highly reactive solvent-intolerant inorganic species.

## Supporting Information Available

Experimental details and data, including ICP results, NMR spectra, and SCXRD structures (PDF)
