# Correction to “Reduction of K^+^ or Li^+^ in the Heterobimetallic Electride K^+^[LiN(SiMe_3_)_2_]e^–^”

**DOI:** 10.1021/jacs.4c09917

**Published:** 2024-08-01

**Authors:** Nathan Davison, Paul G. Waddell, Erli Lu

A key reference^1^ reporting a formal Li^+^ reduction (Figure 1) was omitted in the original Communication. It is cited here along with the reduction described.

**Figure 1:**
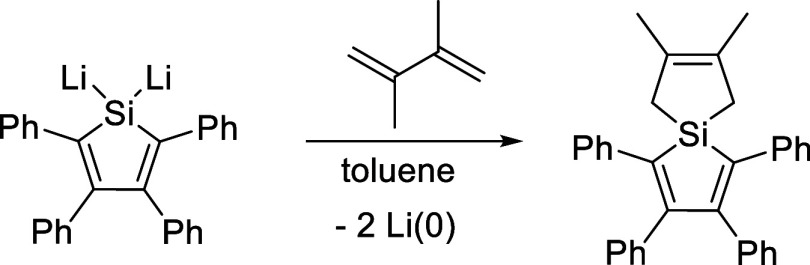
Formal Li^+^ reduction in a 1,1-dilithio-2,3,4,5-tetraphenylsilole.^1^
