# Supplementary material for: Reduction of K+ or Li+ in the Heterobimetallic Electride K+[LiN(SiMe3)2]e–
Source: J Am Chem Soc. 2023 Jul 21;145(31):17007–12. doi: 10.1021/jacs.3c06066 (PMC10416298; doi:10.1021/jacs.3c06066)
Supplement: Supplementary file 1 — ja3c06066_si_001.pdf [file ja3c06066_si_001.pdf]

Reduction of K^+ or Li^+ in a heterobimetallic electride $K^+[LiN(SiMe_3)_2]e^-$

Nathan Davison, Paul G. Waddell, and Erli Lu # *

Chemistry – School of Natural and Environmental Sciences

Newcastle University

Newcastle-upon-Tyne, UK.

*Email of corresponding author (E.L): erli.lu@newcastle.ac.uk

E.L.'s name in simplified Chinese characters: 陆(Lu) 而立(Erli)

Materials and Methods

Section 1. Experimental methods and data

1.1 General procedures
Caution! Quenching Group-1 metal plates (K and Li here) with deionised water is very exothermic and will release highly flammable gas (H_2), hence must be conducted at small scale (<1 mmol) and within a clear fumehood free from any flammable materials (such as solvents, chemicals, tissues). We recommend conducting the quenching in a scintillation vial which is further contained within a sand box. Any large metal pieces must be cut into smaller ones (< 2 mm) in the glovebox before quenching. Appropriate personal protection equipment (such as fire-resistant gloves, safety glasses, lab coat) must be wear throughout the quenching. Any substantial fire must be quenched using fire blanket or a powder fire extinguisher. DO NOT use a CO_2 fire extinguisher!

All manipulations were carried out using in a Vigor™ glovebox equipped with a −35 ˚C freezer and a cold well, under an atmosphere of dry argon. Benzene, *n*-hexane and Et_2O was dried with sodium press, sodium-potassium alloy and distilled under reduced pressure, and kept in the glovebox. Me_6Tren and 2,2,2-cryptand were purchased from Merck and dried under dynamic vacuum for several hours (for 2,2,2-cryptand), or over activated 4Å molecular sieves followed by 3 cycles of freeze-thaw-vacuum degassing (for Me_6Tren), prior to use.

All glassware, including pipettes, vials and ampoules, must be silylated prior to use by treating with trimethylsilyl chloride (Me_3SiCl), rinsing with water, and dried in a 150 °C oven for 12 hours. Failing to silylate glassware will lead to significantly reduced yields, and in some cases, irreproducible results. All the reactions and work-ups herein (bar the water quenching of metals) are conducted in the glovebox in silylate 20 mL scintillation vials.

The organolithium and organosodium complexes in this work are highly reactive towards silicone grease. We would suggest excluding grease as much as possible for all chemicals used herein, including in the solvent distillation apparatus. Otherwise, irreproducible by-products or side products may appear. For this purpose, we used J. Young tap solvent flasks and vacuum transfer apparatus to dry our solvents.

$K^+[LiN(SiMe_3)_2]e^-$ (**1**) was prepared as previously described.[1]

^1H and ^7Li NMR spectra were recorded on a Bruker 300 Avance III spectrometer operating at 300.13, 75.48 and 116.64 MHz respectively. Chemical shifts are quoted in ppm and are relative to $SiMe_4$ (^1H) or external 0.1 M LiCl in D_2O (^7Li).

1.2 Protocol to analyse the metal pieces

The metal pieces obtained from the reactions could be mixtures of Li and K metals. After the reaction, metal pieces are washed with *n*-hexane and Et_2O, dried under vacuum to remove all volatiles, and weighted in the glovebox. Subsequently, the metal pieces are brought out of the glovebox and quenched with deionized water. The aqueous solutions are subject to inductively coupled plasma (ICP) studies, where their Li and K contents are obtained in ppm.

Table S1. The ICP results for deionised water (background) and the metal pieces from the reactions between **1** and Me_6Tren and 2,2,2-cryptand, respectively.

Solution	Metal mass (mg)	K Ave ppm	Li Ave ppm
Deionised water	-	-0.01014	0.001047
Metal from reaction between Me_6tren and **1**	10.1	766.302786	62.935297
Metal from reaction between 2,2,2-cryptand and **1**	10.5	383.063187	128.118925

1.3 Reaction between $K^+[LiN(SiMe_3)_2]e^-$ (**1**) and Me_6Tren

Me_6Tren (0.1152 g, 0.5 mmol) was dissolved in *n*-hexane (1.5 mL). At room temperature, the solution was added to a suspension of $K^+[LiN(SiMe_3)_2]e^-$ (**1**) (0.1032 g, 0.5 mmol) in *n*-hexane (1 ml) in a one-portion manner, which resulted in a mixture of metal plates and blue/grey suspension. The mixture was allowed to stand at room temperature for 15 minutes. Subsequently, the suspension was isolated using a pipette and filtered through a glass wool/Celite plug. The resulting colourless and clear solution was kept at –35 °C to afford $[Li(N(SiMe_3)_2)(Me_6Tren)]$ (**2**) as a colourless crystalline solid (0.1549 g, 78%).

The metal pieces were washed in *n*-hexane (2 × 2 ml) and Et_2O (2 ml) and dried *in vacuo* to afford metal pieces 0.0101 g, which contain K metal 0.255 mmol (51.0% yield based on K from **1**), Li metal 0.0209 mmol (4.2% yield based on Li from **1**) (see Section 1.3.1 for ICP results and detailed deduction).

Single-Crystal X-Ray Diffraction (SCXRD) Study: Single crystals of **2** suitable was grown from n-hexane or Et_2O under –35 °C. **2** is a known complex: our measured cell parameters here match with the previous report [2].

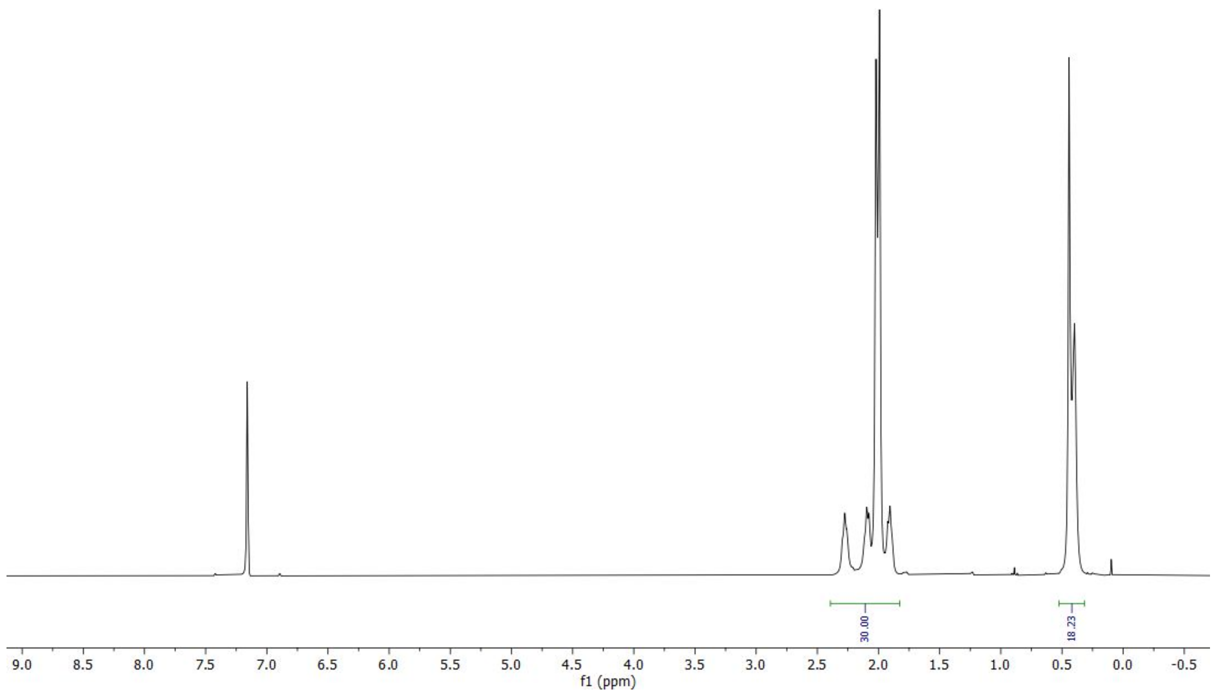

Figure S1: ^1H NMR spectrum (d_6-benzene, 25 °C, 300 MHz) of **2**.

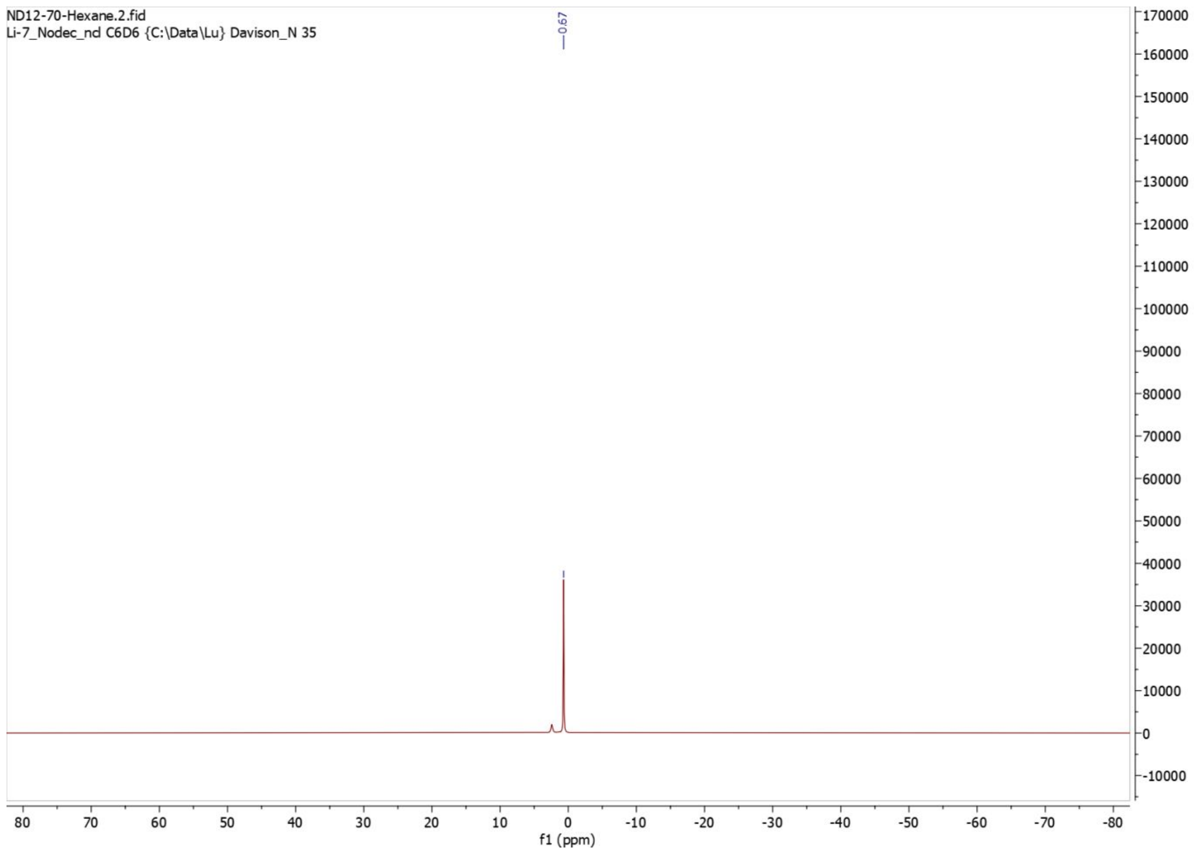

Figure S2: ^{7}Li NMR spectrum (d_6-benzene, 25 ˚C, 117 MHz) of **2**.

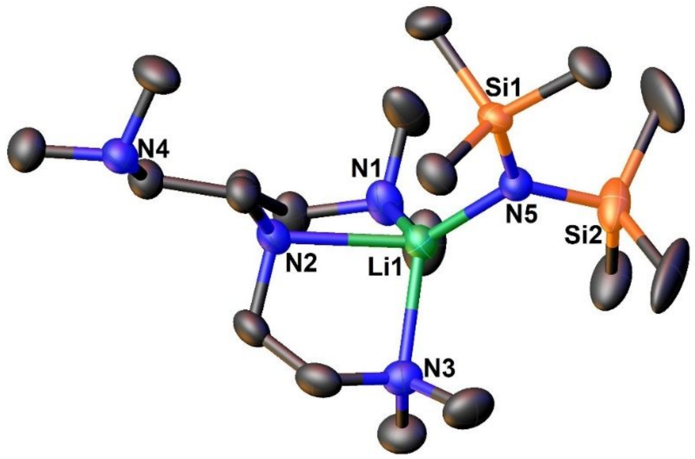

Figure S3: SCXRD Structure of **2**.

1.3.1 ICP Data and deduction for the quenched metal from the reaction between Me_6Tren and **1**

**Solution**	**Metal mass (mg)**	**K Ave ppm**	**Li Ave ppm**
Metal from reaction between Me_6tren and **1**	10.1	766.302786	62.935297

- The overall metal mass: w = 10.1 mg
- The K metal molar percentage in the metallic phase: x = 766.3/(766.3 + 62.9) = 92.4%
- The Li metal molar percentage in the metallic phase: y = 7.6%
- Li metal content in mmol: 0.0209 mmol
- K metal content in mmol: 0.255 mmol
- Li metal yield (based on Li): 0.0209/0.5 = 4.2%
- K metal yield (based on K): 0.255/0.5 = 51.0%

1.4 Reaction between $K^+[LiN(SiMe_3)_2]e^-$ (**1**) and 2,2,2-cryptand

n-Hexane (8 ml) was added to a stirring solid mixture of $K^+[LiN(SiMe_3)_2]e^-$ (**1**) (0.4129 g, 2 mmol) and 2,2,2-Cryptand (0.7530 g, 2 mmol). The resultant mixture was allowed to stir at room temperature for 1 hour. A grey mixture resulted. All volatiles were then removed under vacuum to afford a mixture of grey solids and metal plates. The mixture was extracted by Et_2O (2 × 8 ml) at room temperature. Initially, a mixture of a blue solution, a blue powdery insoluble solid, and metal pieces formed. The blue color of the solution faded within 10 minutes at room temperature, while the blue color of the powdery solid sustains. The suspension (now colorless solution and blue powdery solid) was separated from the metal pieces using a pipette, which then filtered through a glass wool/Celite plug. The three components are treated separately as described below:

(1) After the filtration, the clear solution was kept at –35 °C to afford [K(2,2,2-cryptand)][$N(SiMe_3)_2$] (**3**) as colourless single-crystals that were subject to SCXRD (0.1806 g, 16%). All volatiles were removed from the mother liquor to afford an pale yellow/colorless oil, which was analyzed by ^1H NMR spectroscopy as an intractable mixture.

(2) The blue powdery solid on top of the Celite/glass wool was found to be air-/moisture-sensitive. Further analysis of the powder was hampered by its sensitivity and inseparable mixing with celite/glasswool.

(3) The metal pieces were washed with *n*-hexane (2 ml) and Et_2O (2 × 2ml) and dried *in vacuo* to afford metal pieces 0.0105 g, which contain K metal 0.253 mmol (12.6% yield based on K from **1**), Li metal 0.085 mmol (4.3% yield based on Li from **1**) (see Section 1.4.1 for ICP results and detailed deduction).

SCXRD Study: Single crystals of **3** suitable was grown from Et_2O under –35 °C. **3** is a known complex: our measured cell parameters here match with the previous report [3].

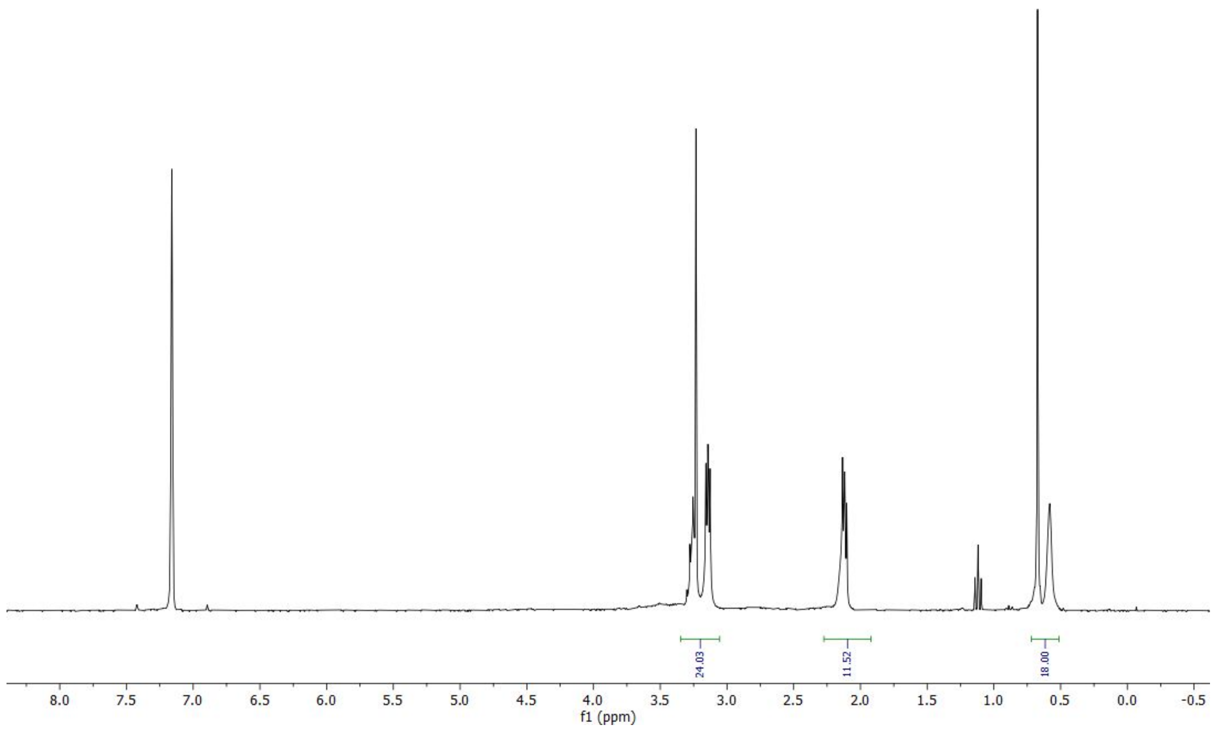

Figure S4: ^{1}H NMR spectrum (d_6-benzene, 25 ˚C, 300 MHz) of **3**.

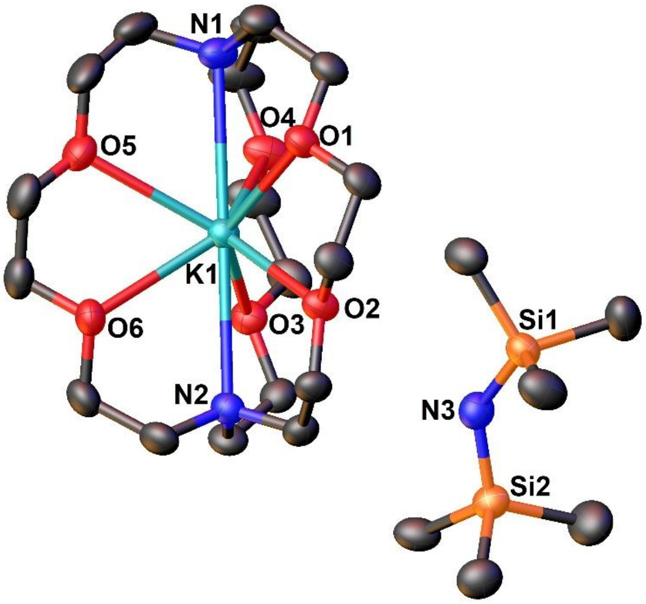

Figure S5: SCXRD Structure of **3.**

1.4.1 ICP Data and deduction for the quenched metal from the reaction between Me_6Tren and **1**

Solution	Metal mass (mg)	K Ave ppm	Li Ave ppm
Metal from reaction between 2,2,2-cryptand and **1**	10.5	383.063187	128.118925

- The overall metal mass: w = 10.5 mg
- The K metal molar percentage in the metallic phase: x = 383.1/(383.1 + 128.1) = 74.9%
- The Li metal molar percentage in the metallic phase: y = 1 - 0.749 = 25.1%
- Li metal content in mmol: 0.085 mmol
- K metal content in mmol: 0.253 mmol
- Li metal yield (based on Li): 0.085/2 = 4.3%
- K metal yield (based on K): 0.253/2 = 12.6%

1.5 Reaction between $K^+[LiN(SiMe_3)_2]e^-$ (**1**) and 18-crown-6

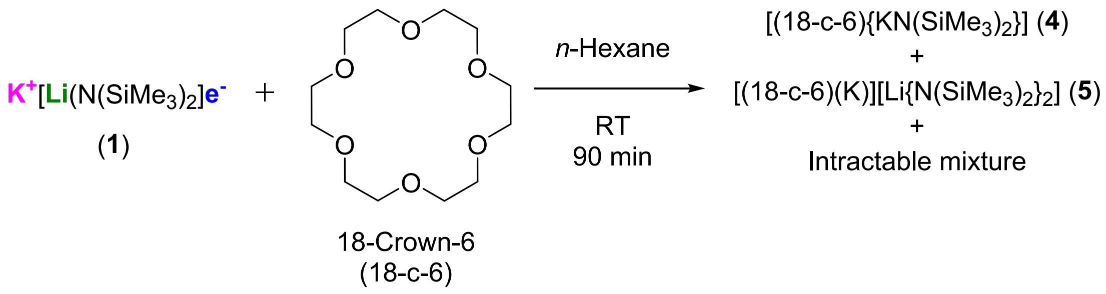

Scheme S1: Reaction between 18-crown-6 and **1**.

At room temperature, a hexane solution of 18-crown-6 (132.0 mg, 0.5 mmol, in 4 mL of *n*-hexane) was dropwisely added into a stirring hexane suspension of **1** (103.2 mg, 0.5 mmol, in 2 mL of *n*-hexane). After the first 2 to 3 drops, the blue color of **1**-hexane suspension suddenly disappeared, to form a colorless, slightly cloudy suspension and a large amount of black slurry. The appearance sustained until all the 18-crown-6 solution was added within 5 minutes. The mixture was then stirred at room temperature for 90 minutes, during which the black slurry solidified into a black solid; while the solution stayed colorless. There was no metal phase during the whole procedure.

All volatiles were remove from the mixture to afford a blackish blue sticky solid as the crude product, which was found undergoes dramatic color change upon contact with d_6-benzene. The crude product (black solid) was initially insoluble in C_6D_6 but suddenly reacted with C_6D_6 and dissolved, forming a deep blue solution. The deep blue color lasted less than 5 seconds at room temperature before converting into a black solution. The crude ^{1}H NMR (collected with the black solution) (Figure S6) is not informative, while the crude ^{7}Li NMR (Figure S7) indicates multiple Li-containing species at this stage.

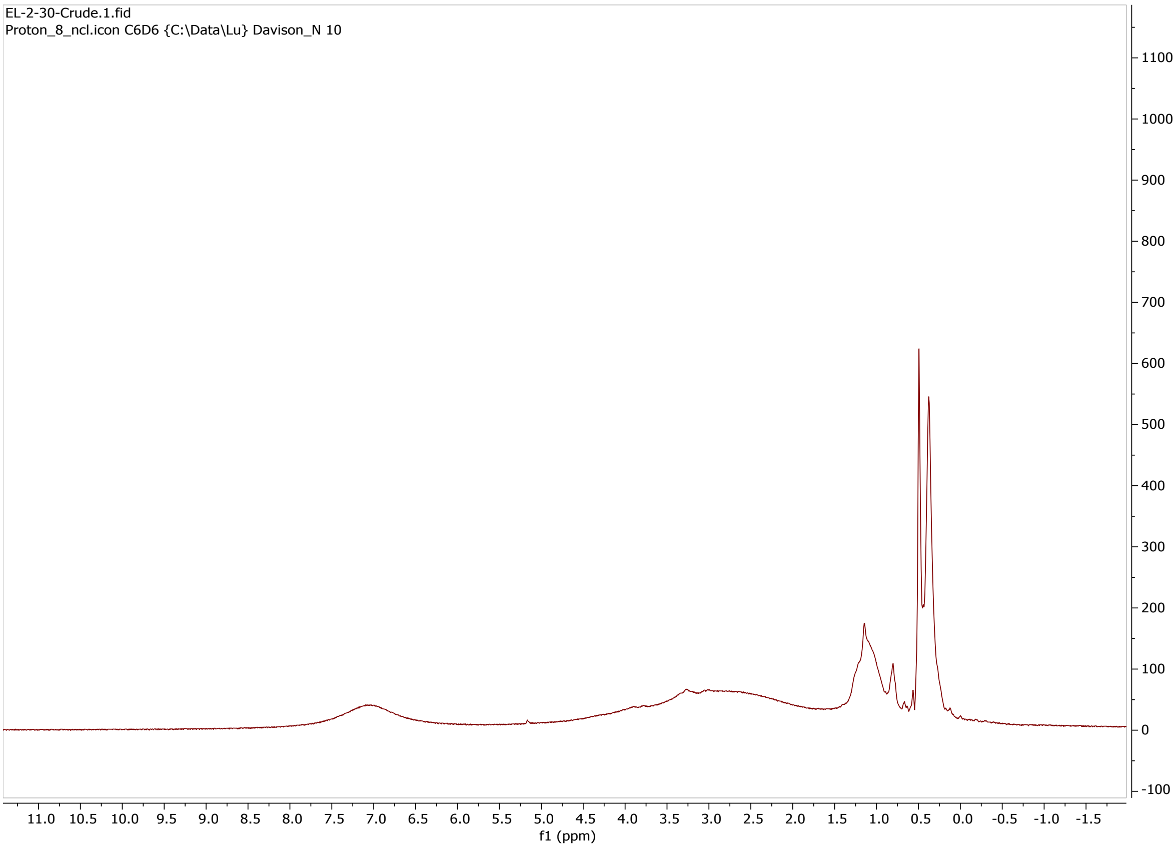

Figure S6. ^{1}H NMR spectroscopy of the crude product (black solid) from the reaction between **1** and 18-crown-6.

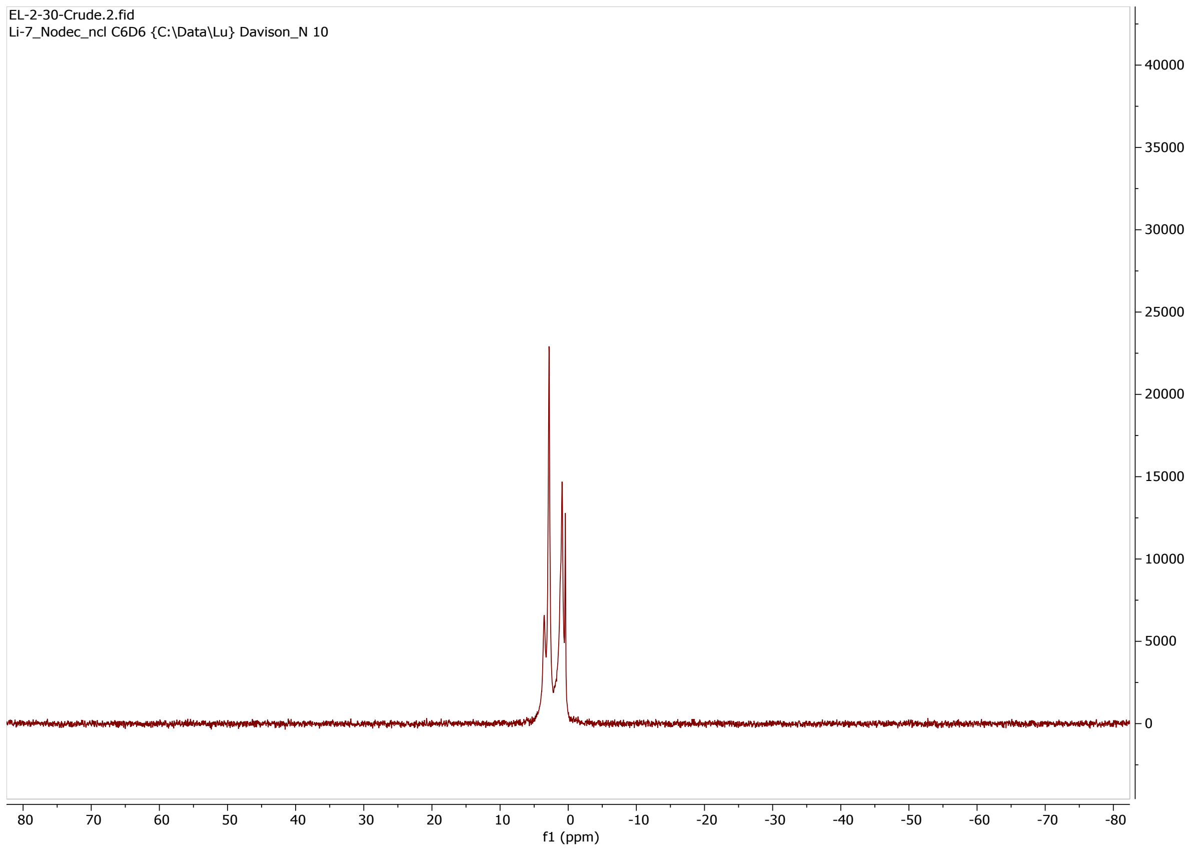

Figure S7. ^{7}Li NMR spectroscopy of the crude product (black solid) from the reaction between **1** and 18-crown-6.

The black crude product was extracted by 3 mL of Et_2O, forming a deep blue solution with very little powdery solid. After filtration, the deep blue solution was kept at -35 ^oC to afford a mixture of crystals of **4** and **5** with a combined mass of 20 mg. It is impossible to isolate **4** and **5** in pure phases due to their similar solubilities, crystallizabilities and crystal habitats. The SCXRD structure of **4** is known,[13] while the SCXRD structure of **5** is displayed in Figure S8.

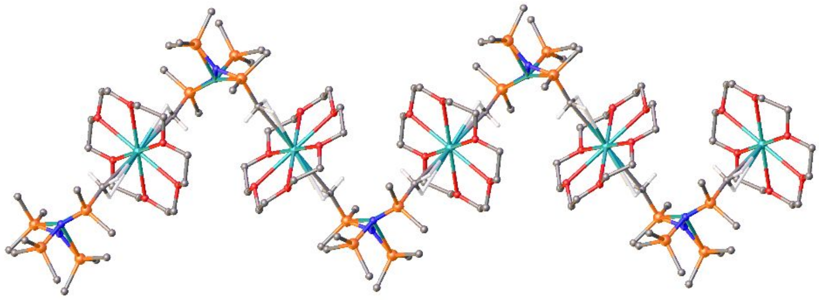

Figure S8. SCXRD structure of **5**.

References

1. Davison, N.; Quirk, J. A.; Tuna, F.; Collison, D.; McMullin, C. L.; Michaels, H.; Morritt, G. H.; Waddell, P. G.; Gould, J. A.; Freitag, M.; Dawson, J. A.; Lu, E. *Chem* **2023**, *9*, 576-591.
2. Cousins, D. M.; Davidson, M. G.; Frankis, C. J.; García-Vivó, D.; Mahon, M. F. *Dalton Trans.* **2010**, *39*, 8278-8280.
3. Xu, M. –T.; Jupp, A. R.; Qu, Z. –W.; Stephan, D. W. *Angew. Chem. Int. Ed.* **2018**, *57*, 11050-11054.
4. C. Belger, N. M. Neisius and B. Plietker, *Chem. -Eur. J.*, 2010, **16**, 12214-12220.
5. Y. Yokoyama, H. Nagashima, S. M. Shrestha, Y. Yokoyama and K. Takada, *Bull. Chem. Soc. Jpn.*, 2003, **76**, 355-361.
6. X.-H. Ouyang, R.-J. Song, B. Liu and J.-H. Li, *Adv. Synth. Catal.*, 2016, **358**, 1903-1909.
7. M. Matsui, M. Tsuge, K. Shibata and H. Muramatsu, *Bull. Chem. Soc. Jpn.*, 1994, **67**, 1753-1755.
8. L. Davin, A. Hernán-Gómez, C. McLaughlin, A. R. Kennedy, R. McLellan and E. Hevia, *Dalton Trans.*, 2019, **48**, 8122-8130.
9. C. Brinkmann, A. G. M. Barrett, M. S. Hill and P. A. Procopiou, *J. Am. Chem. Soc.*, 2012, **134**, 2193-2207.
10. Y. Zhao, Y. Zhou, J. Liu, D. Yang, L. Tao, Y. Liu, X. Dong, J. Liu and J. Qu, *J. Org. Chem.*, 2016, **81**, 4797-4806.
11. M. Schäfer, T. Stünkel, C. G. Daniliuc and R. Gilmour, *Angew. Chem. Int. Ed.*, 2022, **61**.
12. K. Bojaryn, S. Fritsch and C. Hirschhäuser, *Org. Lett.*, 2019, **21**, 2218-2222.
13. The SCXRD structure of **4** has the CCDC codes XUSTOU, XUSTOU01 and XUSTOU02.
